# *Coccoloba alnifolia* Leaf Extract as a Potential Antioxidant Molecule Using In Vitro and In Vivo Assays

**DOI:** 10.1155/2020/3928706

**Published:** 2020-10-10

**Authors:** Luciana Fentanes Moura de Melo, Dayanne Lopes Gomes, Lucas Felipe da Silva, Larissa Marina Pereira Silva, Marina Lopes Machado, Cesar Orlando Muñoz Cadavid, Silvana Maria Zucolotto, Riva de Paula Oliveira, Deborah Yara Alves Cursino dos Santos, Hugo Alexandre Oliveira Rocha, Katia Castanho Scortecci

**Affiliations:** ^1^Laboratório de Transformação de Plantas e Análise em Microscopia (LTPAM), Departamento de Bioquímica, Universidade Federal do Rio Grande do Norte (UFRN), Natal 59078-970, Brazil; ^2^Laboratório de Biotecnologia de Polímeros Naturais (BIOPOL), Departamento de Bioquímica, Universidade Federal do Rio Grande do Norte (UFRN), Natal 59078-970, Brazil; ^3^Instituto Federal de Educação, Ciência e Tecnologia do Piauí (IFPI), Campus de São Raimundo Nonato, São Raimundo Nonato 64770-000, Brazil; ^4^Laboratório de Produtos Naturais e Bioativos (PNBio), Departamento de Farmácia, Universidade Federal do Rio Grande do Norte (UFRN), Natal 59010-180, Brazil; ^5^Laboratório de Genética Bioquímica (LGB), Departamento de Biologia Celular e Genética, Universidade Federal do Rio Grande do Norte (UFRN), Natal 59078-970, Brazil; ^6^Programa de Pós-graduação em Ciências Biológicas, Bioquímica Toxicológica, Departamento de Bioquímica e Biologia Molecular, Centro de Ciências Naturais e Exatas, Universidade Federal de Santa Maria, Camobi, 97105-900 Santa Maria, RS, Brazil; ^7^Laboratório de Fitoquímica, Departamento de Botânica, Universidade de São Paulo (USP), São Paulo, SP 05508-090, Brazil

## Abstract

The genus *Coccoloba* is widely used in traditional folk medicine, but few scientific data exist for this genus. The goal of this study was to characterise the chemical composition and antioxidant activities of *C. alnifolia* leaf extracts using *in vitro* and *in vivo* assays. Six extracts were obtained: hexane (HE), chloroform (CE), ethanol (EE), methanol (ME), water end extract (WEE), and water extract (WE). Thin-layer chromatography (TLC) analysis showed the presence of phenols, saponins, terpenes, and flavonoids. *In vitro* assays demonstrated substantial antioxidant potential, especially for polar extracts (EE, ME, WEE, and WE). Moreover, no toxic effects were observed on mammalian cell lines for most of the extracts at the concentrations evaluated. The nematode *Caenorhabditis elegans* was also used as an *in vivo* model for testing antioxidant potential. The EE and WE were chosen, based on previously obtained results. It was observed that neither the EE nor the WE had any toxic effect on *C. elegans* development. Additionally, the antioxidant potential was evaluated using *tert*-butyl hydroperoxide as a stressor agent. The EE increased the life span of *C. elegans* by 28% compared to that of the control, and the WE increased the range to 39.2-41.3%. High-performance liquid chromatography (HPLC-DAD) showed the presence of gallic acid, *p*-coumaric acid, and vitexin in the WE. Therefore, *in vitro* and *in vivo* data demonstrated the antioxidant potential of *C. alnifolia* extracts and their possible biotechnological applications.

## 1. Introduction

Free radicals are produced as a normal part of metabolism by mitochondria, peroxisomes, phagocytosis, inflammatory processes, ischaemia, and physical exercise [1]. The balance between the production and neutralisation of reactive oxygen species (ROS) by antioxidant systems is very important, and an imbalance tends towards ROS overproduction leading to so-called oxidative stress [2, 3].

Additionally, cells are normally targets of reactive species such as reactive oxygen species (ROS), reactive nitrogen species (RNS), and reactive sulfur species (RSS). This imbalance may damage molecules such as proteins, lipids, DNA, and RNA and lead to metabolic disorders and diseases such as cancer, cardiovascular diseases, diabetes, atherosclerosis, stroke, neurological disorders, renal disorders, liver disorders, hypertension, and rheumatoid arthritis, among others associated with oxidative stress [2–5].

Plants produce a mixture of compounds known as secondary metabolites that have different biological and pharmacological properties. These secondary metabolites are known to have antioxidant potential that is important for maintaining this oxidative balance. These molecules may be obtained from different plant tissues such as leaves, bark, roots, and fruit [6]. Thus, the identification and isolation of compounds are important research topics in this field [7].

The Polygonaceae (Caryophyllales) has approximately 51 genera and 1100 species distributed throughout various biomes worldwide. Some species are used as ornamental plants, some are cultivated for medicinal purposes, and some are economically important for the supply of wood for the production of household items [8]. The genus *Coccoloba* consists of approximately 46 species. Of these, 21 are endemic to Brazil and distributed in several biomes: Amazon Forest, Atlantic Forest, Caatinga, Brazilian savanna (Cerrado), and Pantanal. *Coccoloba* plants have articulated stems, alternate and whole simple leaves, with concresced stipules, and arteries. They are herbaceous, shrubby, arboreal, or lianas [9].

The chemical composition for *Coccoloba* has been described; it contains triterpenes, diterpenes, anthraquinones, phytosteroids, alkaloid benzenoids, saponins, flavonoids, tannins, gallic acid, epigallocatechin, gallate, myristin-3-O-8-rhamnoside, *β*-sitosterol, *β*-lupeol, ursolic and betulic acids, carboxylic acids, esters, aldehydes, ellagic acid, benzoic acid, *o*-coumaric acid, rutin, myricetin, and quercetin [10–12]. The species *C. uvifera*, *C. cereifera*, and *C. mollis* have been evaluated as larvicidal agents against *Aedes aegypti* mosquitoes [13], as well as antifungal [14], cytotoxic [15], antimicrobial [10], wood-biofungicidal, and phytopatogenic bacterial [12].

*Coccoloba alnifolia*, popularly known as “pau-de estalo” or “cabuçu,” is one of the endemic Brazilian species of this genus. Despite all these uses in traditional folk medicine, there is no scientific information about the secondary metabolite composition of *C. alnifolia* nor about their possible biological activities. The goal of this study was to characterise the chemical composition and the antioxidant activities of *C. alnifolia* extracts using *in vitro* and *in vivo* models. Five leaf extracts were obtained through a serial extraction (using apolar solvents to polar solvents), and the sixth extract was made with only water (based on folk use). In general, these extracts did not affect the viability of six different mammalian cell lines. In addition, *in vitro* and *in vivo* assays showed four polar extracts, EE, ME, WEE, and WE, which were sources of antioxidant molecules. Moreover, EE and WE extracts did not affect the fecundity of *Caenorhabditis elegans* nematodes used as a test model. Therefore, *Coccoloba alnifolia* leaves have excellent potential for the development of herbal medicines and as antioxidant products.

## 2. Materials and Methods

### 2.1. Reagents

Potassium ferricyanide, ferrous sulfate II, trichloroacetic acid, and sulfuric acid were purchased from Merck (Darmstadt, Germany). Nitro blue tetrazolium (NBT), monosaccharides, diaminoethanetetraacetic acid (EDTA), D-glucose, acid gallic, ascorbic acid, bovine serum albumin protein, ascorbic acid, methionine, 3-(4,5-dimethylthiazolyl-2)-2,5- diphenyltetrazolium bromide (MTT), pyrocatechol violet, riboflavin, ammonium molybdate, and tert-butyl hydrogen peroxide (t-BOOH) were purchased from Sigma-Aldrich Co. (St. Louis, MO, USA). Sodium bicarbonate, nonessential amino acids, and phosphate-buffered saline (PBS) were purchased from Invitrogen Corporation (Burlington, ON, Canada). Dulbecco's Modified Eagle Medium (DMEM) and fetal bovine serum (FBS) were obtained from CULTILAB (Campinas, SP, Brazil). Penicillin and streptomycin were obtained from Gibco (Fort Worth, TX, USA). All other solvents and chemicals were of analytical grade from Synth (Diadema, SP, Brazil).

### 2.2. Plant Material

*Coccoloba alnifolia* mature leaves were harvested on May 2015 from a specimen located at the Parque das Dunas, Natal, RN, Brazil (UTM zone 250257315 m E 9357108 m N—GPS Garmin Etrex). This region corresponds to the Atlantic Forest biome. After identification, a voucher was deposited in the UFRN Herbarium with number: UFRN 17133. The research was registered at SISBIO no. 54064-1, SISGEN no. A39FD4C.

### 2.3. Preparation of Extracts

After harvest, fresh leaves were divided into small pieces and transferred into a flask at a proportion of 1 : 10 (*w*/*v*) (100 g fresh leaves to 1000 mL of solvent) to be macerated during 24 h for each solvent used. A serial extraction approach was made using different solvents following the order from apolar to polar solvents like hexane (HE), chloroform (CE), ethanol (EE), methanol (ME), and water end extract (WEE). The approach used to prepare the extracts was a serial extraction using different solvents where the leaves were macerated, and it was added each solvent (apolar to polar solvents), for 24 h. Then, leaves were filtered and moved back to the flask, and the next solvent was added. In this way, water end extract (WEE) corresponds to the last solvent used for this serial extraction. The idea of a serial extraction separated the compound based on the solvent. Furthermore, the extract made using only water—WE—was based on the folk used.

To prevent light degradation, the flask was covered with aluminum foil. Then, it was placed on a shaker table at 150 rpm for 24 h at 24°C (Tecnal Shacker). The extract was filtered through Whatman No. 1 paper. Leaves were transferred back to the flask with the next solvent following the seriated extraction under the same conditions. The extracts were dried in a rotaevaporator (Tecnal–TE 210) at 40°C. The extracts were recovered into 1% DMSO (Merck), then lyophilized (Labconco FreeZone 4.5). All extracts were resuspended into water at a final concentration of 100 mg/mL (stock extract) and kept at -18°C until use.

### 2.4. Total Content of Sugar, Phenolic Compounds, and Protein

#### 2.4.1. Total Sugars

To verify the amount of sugars in the extracts, the phenol-H_2_SO_4_ method was used and the D-glucose (Sigma-Aldrich) as a standard. The total sugars were measured at 490 nm (Hitachi U-2000 Tokyo, Japan) [16].

#### 2.4.2. Total Phenolic Compounds

The content of total phenolic compounds was measured using Folin Ciocalteu method, and gallic acid (Sigma-Aldrich) was used as standard. It was measured at 765 nm (BioTek Epoch Microplate, California, CA, USA) [17].

#### 2.4.3. Total Soluble Proteins

Total protein content was measured using Bradford method, and bovine serum albumin protein (Sigma-Aldrich) was used as standard. The reaction was measured at 595 nm (BioTek Epoch Microplate, California, CA, USA) [18].

### 2.5. In Vitro Antioxidant Activity

Each antioxidant assay was done in triplicates and repeated three times. All readings were done using the spectrophotometer BioTek Epoch Microplate (California, CA, USA). The extracts from *C. alnifolia* were used at 250 *μ*g/mL.

#### 2.5.1. Total Antioxidant Capacity (TAC)

The antioxidant activity was measured considering the reduction of Mo^+6^ to Mo^+5^ by the plant extracts and subsequent formation of a green phosphate/Mo^+5^ complex at acid pH. The extract concentration used was 250 *μ*g/mL that was mixed to the 600 mM sulfuric acidammonium molybdate), and it was incubated at 100°C for 90 min [19]. After this time, the mixture was kept at room temperature to cool down, and the absorbance was measured at 695 nm. The results were compared to the negative control (distilled water). The total antioxidant capacity was expressed in equivalents of ascorbic acid (EAA/g).

#### 2.5.2. DPPH

The antioxidant activity was determined by the ability of the antioxidants present in the samples to scavenging the radical DPPH [20]. The extract was added at concentration of 250 *μ*g/mL and 100 *μ*L of DPPH (0.1 mM). The mixture was mixed vigorously, and it was allowed to stand at room temperature during 30 min. Then, the absorbance was then measured at 517 nm. The blank control was ethanol 99.5% (Synth, Brazil), and the negative control was distilled water. The DPPH scavenging activity was calculated as follows: Scavenging activity (%) = [(1 − (sample − blank))/negative control] × 100%.

#### 2.5.3. Reducing Power Assay

The reducing power of the samples were evaluated by the reduction of potassium ferricyanide into potassium ferrocyanide [21]. The plant extract at 250 *μ*g/mL was added to a solution containing 0.2 M phosphate buffer (pH 6.6) and potassium ferricyanide (1% *w*/*v*) in a final volume of 4 mL. The reaction was incubated at 50°C for 20 min; after this period, it was stopped by adding the TCA solution (10% *w*/*v*). The solution was then mixed with distilled water and ferric chloride (0.1% *w*/*v*). The absorbance was measured at 700 nm. Phosphate buffer was used as blank control. The result was expressed as a percentage of the activity presented by 0.1 mg/mL ascorbic acid (standard—Sigma-Aldrich).

#### 2.5.4. Superoxide Scavenging Activity

The assay is based on the ability from the extracts to inhibit the photochemical reduction of nitroblue tetrazolium (NBT) into riboflavin-light-NBT system [21, 22]. To do this, the extract at concentration of 250 *μ*g/mL was added into 50 mM phosphate buffer (pH 7.8), 13 mM methionine, 100 mM EDTA, 75 mM NBT, and 2 mM riboflavin. After, the mixture, the solution was exposed for 10 minutes to a fluorescent lamp. The change of color to blue was due to formazan production, and it was monitored by absorbance at 560 nm. In the spectrophotometer. EDTA was used as a control and distilled water as blank. Results were expressed as percent scavenging:

(1) $$\begin{array}{c} \text{Percentage of superoxide scavenging}=\left(\left[\text{standard control}-\text{sample}\right]/\left[\text{standard control}-\text{blank}\right]\right)\times 100. \end{array}$$

### 2.6. Cell Viability—MTT Assay

The effect of the *C. alnifolia* extracts was evaluated on cell viability. One nontumour cell line was used: NIH/3T3 (ATCC CRL-1658—a murine fibroblast) as well as five tumour cell lines: HeLa (ATCC CCL-2—an adenocarcinoma cell of human uterus), SiHa (ATCC HTB-35—a human squamous cellular carcinoma cell), PC-3 (ATCC CRL-1435—a human prostate adenocarcinoma cell), B16-F10 (ATCC CRL-6475—a mouse skin melanoma cell), and PANC-1 (ATCC CRL-1469—a pancreatic adenocarcinoma cells). First, cell lines were grown in culture flasks using the Dulbecco's Modified Eagle Medium (DMEM) supplemented with FBS (10% *v*/*v*) and antibiotics (100 U/mL penicillin and 100 *μ*g/mL streptomycin). These culture flask were maintained in a humidified 5% CO_2_ atmosphere at 37°C. To evaluate the extract effect on cell viability, the cells were transferred into 96-well plates at 5 × 10^3^ cell per well until they reach the confluence. The extracts (HE, CE, EE, ME, WEE, and AE) were added at 0, 100, 250, or 500 *μ*g/mL for 24 h at 37°C in a 5% CO_2_ atmosphere. After the incubation period, the medium was changed to 100 *μ*L of MTT (1 mg/mL dissolved in DMEM). Then, cells were incubated for 4 h in a 5% CO_2_ atmosphere at 37°C. Next, the wells were aspirated, and formazan crystals were solubilized by the addition of 100 *μ*L/well ethanol [23, 24]. Plate was read at 570 nm absorbance using an Epoch microplate spectrophotometer (BioTek Instruments Inc., Winooski, VT, USA). Cell viability was determined and compared to the negative control (only DMEM) using the following formula: %viability = (*A*_test_/*A*_control_) × 100, in which *A*_test_ corresponded to the absorbance of the experimental group, and *A*_control_ was the absorbance of the negative control.

### 2.7. In Vivo Antioxidant Activity

#### 2.7.1. *Caenorhabditis elegans*—Maintenance and Treatment

*C. elegans* N2 (wild-type line) were obtained from the *Caenorhabditis* Genetics Center (University of Minnesota, USA) and was maintained in nematode growth medium (NGM) seeded with *Escherichia coli* OP50 at 20°C according to Brenner [25]. Worms were synchronized using bleaching solution in gravid hermaphrodites (NaOCl 1%, NaOH 0.25 M) to have animals at L1 stage (Sulston and Hodgkin, 1988). For treatment, EE and WEE extracts were filtered using a 0.22 *μ*m filter (Merck) and added to the NGM at final concentrations of either 1 or 10 mg/mL. Synchronized L1 were cultivated on NGM plates containing extracts and seeded with *E. coli* OP50 for 48 h at 20°C until they reached L4 stage.

#### 2.7.2. Toxicity on *Caenorhabditis elegans* Eggs

For this assay, 30 eggs were placed into three NGM plates containing EE and WEE at 0, 1, and 10 mg/mL and kept at 20°C for 48 h. After this, the number of L4 and eggs were counted. This assay was done in three independent assays having a total number of 90 eggs/group.

#### 2.7.3. Oxidative Stress Assay

The oxidative stress assay was performed using tert-butyl hydrogen peroxide (t-BOOH) as a ROS producer [26, 27]. Forty worms at L4 stage were treated with EE and WEE at concentrations 0, 1, and 10 mg/mL for 48 h at 20°C. After that, worms were transferred to 12-well plate having M9 buffer with 8 mM t-BOOH and evaluated every half hour. Those worms that did not react to the stimulus were considered dead (no movement). Some worms were censored, which means when the worm was not found or it died for reasons other than the oxidative stress. These analyses were done in five independent assays.

### 2.8. Thin-Layer Chromatography (TLC)

Chromatoplates of silica gel *F*_254_ were used for the analyses. The mobile phases used were as follows: (i) ethyl acetate : formic acid : water (8 : 0.8 : 1, *v*/*v*/*v*), (ii) ethyl acetate : formic acid : methanol : water (10 : 0.5 : 0.6 : 0.2, *v*/*v*/*v*/*v*), and (iii) toluene : ethyl acetate : formic acid (5 : 5 : 0.5, *v*/*v*/*v*). Compound visualization was achieved by spraying solutions of sulfuric vanillin, 0.5% Natural A Reagent (difenilboriloxietilamina), 1% ferric chloride in methanol, and Dragendorff. The standards used at TLC were as follows: quercetin, ursolic acid, gallic acid, isoquercetin, chlorogenic acid, ellagic acid, isoquercitrin, luteonin, canferol, rutin, caffeic acid, catechin, orientin, isoorientin, vitexin, and isovitexin (Sigma-Aldrich purity ≥ 95%). Then, the color was observed, and the retention factor (Rf) of spots measured and compared with literature [28]. For the ME and EE, a Co-TLC was done to confirm the presence of glycoside flavonoids vitexin and isovitexin.

### 2.9. High-Performance Liquid Chromatography (HPLC-DAD)

The WE was analysed by HPLC-DAD (Agilent 1260) equipped with a C18 column (Zorbax—150 mm × 4.6 mm i.d.×3.5 *μ*m). The elution was done with a gradient of 0.1% acetic acid (A) and acetonitrile (B) as follows: 10% B (0-6 min), 10 to 15% B (6-7 min), 15% B (7-22 min), 15 to 50% B (22-32 min), 50 to 100% B (32-42 min), and 100% B (42-50 min), at a constant flow rate of 1 mL/min. The sample was prepared at 1 mg/mL using methanol (HPLC grade), and the volume of injection was 3 *μ*L. The column temperature was 45°C, and detection was done at 228, 254, 280, 290, 320, 340, and 352 nm. The identification of phenolic compounds was obtained by comparison of retention time and UV-visible absorption spectra with standard compounds.

The standards used in the HPLC-DAD were as follows: 3-hydroxycinnamic acid (501-52-0), benzoic acid (65-85-0), caffeic acid (331-39-5), cinnamic 92 acid (621-82-9), chlorogenic acid (327-97-9), ellagic acid (476-66-4), ferulic acid (1135-93 24-6), gallic acid (149-91-7), gentisic acid (490-79-9), o-coumaric acid (583-17-5), p-coumaric acid (501-98-4), rosmarinic acid (20283-92-5), sinapic acid (530-59-6), apigenin (520-36-5), apiin—apigenin-7-(2-O-apiosylglucoside) (26544-34-3), kaempferol (520-18-3), kaempferol 3-O-D-galactoside (23627-87-4), 3-O-methylkaempferol (1592-70-7), biorobin—kaempferol 3-O-*β*-robinobioside (17297-56-2), nicotiflorin—kaempferol 3-O-*β* rutinoside (17650-84-9), catechin (154-23-4), chrysin—5,7-dihydroxyflavone (480-40-0), chrysoeriol—3′-O-methylluteolin (491-71-4), daidzein—4,7-dihydroxyisoflavone (486-66-8), epicatechin (490-46-0), genistein—4′,5,7-trihydroxyisoflavone (446-72-0), gossypetin—8-hydroxyquercetin (489-35-0), hesperidin (520-26-3), hesperitin (520-33-2), hispidulin—6-methoxyapigenin (1447-88-7), homoorientin—luteolin 6-C-*β*-D glucoside (4261-42-1), isorhamnetin 3-O-*β*-galactoside (6743-92-6), isorhamnetin 3-O-*β*-D-glucoside (5041-82-7), keioside—isorhamnetin 3-O-robinobioside (107740-46-5), narcissin—isorhamnetin 3-O-*β* rutinoside (604-80-8), luteolin (491-70-3), myricetin (529-44-2), naringenin (480-41-1), neohesperidin (13241-33-3), orientin—luteolin 8-C-glucoside (28608-75-5), quercetagetin-7-O-glucoside (548-75-4), quercetin (117-39-5), hyperin—quercetin 3-O-*β*-galactoside (482-36-0), isoquercetrin—quercetin 3-O-*β*-glucoside (482-35-9), quercetin 3-O-*β*-gentiobioside (7431-83-6), quercetin 3-O-robinobioside (52525-35-6), quercitrin—quercetin 3-O-rhamnoside (522-12-3), rhamnetin (90-19-7), rutin—quercetin 3-O-*β*-rutinoside (153-18-4), taxifolin—dihydroquercetin (480-18-2), tiliroside—kaempferol 3-O-(6^″^-O-p-coumaroyl) glucoside (20316-62-5), and vitexin—apigenin 8-C114 glucoside (3861-93-4). Other standards correspond to the library made in the Phytochemistry Lab, Department of Botany, University of São Paulo.

### 2.10. Bioinformatics Analysis

Through TLC analyses, the presence of two glycoside flavonoids vitexin and isovitexin in *C. alnifolia* leaf extracts was observed. These compounds were used to search at the Traditional Chinese Medicine System Pharmacology (TCMSP) database for gene targets [29]. The data obtained at TCMSP were used at the Kyoto Encyclopedia Genes and Gnome (KEGG) database [30]. The pathways identified at KEGG were listed in descending order according to *p* value. These data were used to build a network using String 10 version 11 (https://string-db.org/).

### 2.11. Statistical Analysis

All results were expressed as mean ± standard. Each assay was done as a triplicate or a quintuplet, and each one was repeated at least 3 times. Statistical analysis was done using the GraphPad Prism 6.0 (GraphPad software Inc., San Diego, CA, USA), using the variation analysis (one-way ANOVA) and Tukey's post-test to compare the different groups. Differences that had *p* values ≤ 0.05 were considered significant.

## 3. Results

### 3.1. Total Content of Sugar, Phenolic Compounds, and Protein

Five extracts were obtained in a series from nonpolar to polar: hexane (HE), chloroform (CE), ethanol (EE), methanol (ME), and water end extract (WEE), and the sixth was made only with water—water extract (WE).  Table 1 shows that the total phenolic compounds ranged from 2.3 *μ*g/g to 61.26 *μ*g/g, in order HE < CE < WE < WEE < EE < ME. The sugar content ranged from 33.45 *μ*g/g to 225.00 *μ*g/g, and the order was CE < HE < WEE < EE < WE < ME. The protein content was low ranging from 1.1 to 4.5 *μ*g/g.

### 3.2. *In Vitro* Antioxidant Activity

Considering the presence of phenolic compounds and understanding that these phytochemicals may be associated with antioxidant activity, the antioxidant potential of each of the six *C. alnifolia* extracts was analysed using four assays: total antioxidant capacity (TAC), DPPH (2,2-diphenyl-1-picryl-hydrazyl-hydrate) free radical method, reducing power test, and superoxide scavenging activity.

**Figure 1:**
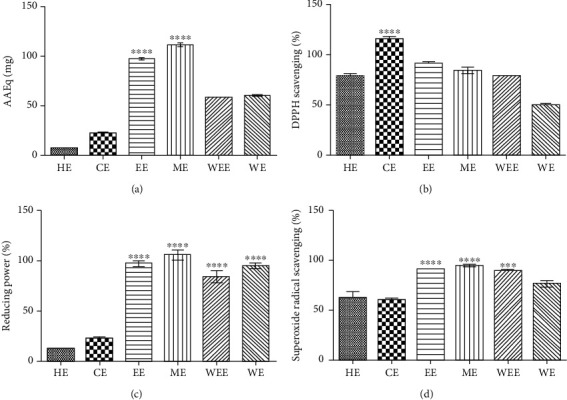
*In vitro* antioxidant activities using *Coccoloba alnifolia* extracts. (a) Total antioxidant capacity (TAC). The *x*-axis corresponds to different extracts, and the *y*-axis corresponds to the ascorbic acid equivalents in mg (AAEq mg). (b) Scavenging of the DPPH radical. The *x*-axis corresponds to different extracts used, and the *y*-axis corresponds to the percentage of DPPH scavenging. (c) Reducing power. The *x*-axis corresponds to different extracts, and the *y*-axis corresponds to the percentage of antioxidant activity. (d) Superoxide radical scavenging. The *x*-axis corresponds to different extracts, and the *y*-axis corresponds to percentage of antioxidant activity. For each assay the extract concentration used was 250 *μ*g/mL. HE: hexane extract; CE: chloroform extract; EE: ethanol extract; ME: methanol extract; WEE: water end extract; WE: water extract. Each assay was done in triplicate, and the assays were three times. The results were analyzed using an ANOVA and Tukey's test. ^∗∗∗∗^Significant difference between extracts at *p* < 0.001; ^∗∗∗^Significant difference between the extracts at *p* < 0.01.

The TAC assay evaluated the ability of the sample to donate electrons and thus neutralise reactive species. The highest values of TAC were found for ME (111.38 EEAq mg) and EE (96 EEAq mg). WEE (58.25 EEAq mg) and WE (59.89 EEAq mg) presented equivalent results (Figure 1(a)). For the DPPH assay, the antioxidant activity ranged from 49% to 114% (Figure 1(b)). Moreover, polar extracts showed the highest activities for the reducing power assay, for which the values ranged from 83.9% to 105.4% (Figure 1(c)). A similar result was also observed for superoxide radical scavenging assay, where the highest values were observed for polar extracts, and these values ranged from 76.4% to 91% (Figure 1(d)).

Pearson coefficient analyses showed a positive correlation between the phenolic compound content of the *C. alnifolia* extracts and the antioxidant assays. There was a strong correlation between TAC and reducing power assay (Sup. Table 1), and a moderate correlation with superoxide radical scavenging. Furthermore, the sugar content presented a very strong correlation for reducing power assay and TAC and a moderate correlation with superoxide radical scavenging (Sup. Table 1).

### 3.3. *In Vivo* Analysis

The data from the antioxidant assays described above prompted us to perform antioxidant assays *in vivo*. However, before carrying out these assays, it was necessary to assess the toxic potential of these extracts to rule out a possible harmful effect on the animals used in the *in vivo* experiments. Then, their effects on the nontumour cells 3T3 and five tumour cell lines, HeLa, SiHa, PC-3, B16-F10, and PANC-1, were analyzed. After that, *Caenorhabditis elegans* was used as the *in vivo* model.

#### 3.3.1. Cell Viability

For the cell lines, the effect of the six extracts on three concentrations (100 *μ*g/mL, 250 *μ*g/mL, and 500 *μ*g/mL) were evaluated (Figure 1). These extracts did not have any effect on cell viability at any of the three concentrations analysed. However, for the ME and WEE, a reduction in cell viability for nontumour cells of approximately 25%-35% was observed (ME at 250 or 500 *μ*g/mL and for WEE at 500 *μ*g/mL) (Table 2). Furthermore, for tumour cell lines, in general, there was no significant change in cell viability. A reduction in cell viability ranging 10-25% for HE, CE, and WE was observed for HeLa and SiHa cell lines (Table 2). Thus, in general for nontumour and tumour cell lines, *C. alnifolia* extracts were not cytotoxic to the *in vivo* cell line model.

#### 3.3.2. Toxicity of *C. elegans* Eggs

**Figure 2:**
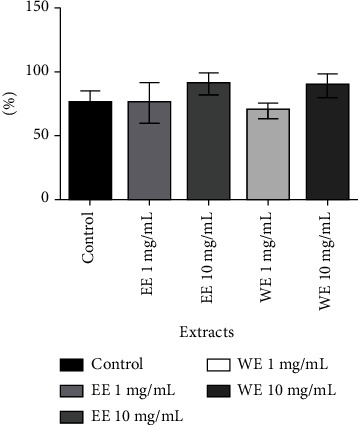
Percentage of the hatched eggs of *C. elegans* treated with extracts of *C. alnifolia*. The *x*-axis corresponds to the treatments with ethanol (EE) and water extract (WE) at 0 mg/mL (control), 1 mg/mL, and 10 mg/mL. The *y*-axis corresponds to the percentage egg hatching. Data were obtained in quintuplicate, and they were analysed by an ANOVA and Tukey's test with significance set at *p* ≤ 0.05.

*Caenorhabditis elegans* has an impermeable cuticle, which is an extracellular matrix of collagen, forming a multifunctional exoskeleton, which makes the passage of substances difficult [31]. Because of this, higher extract concentrations were used compared to cell line assays. Considering the results obtained with antioxidants and with cell lines, the extracts, EE and WE, were selected for evaluation. For this model, 1 and 10 mg/mL extract concentrations were used to evaluate the possible toxic effect on egg hatching (Figure 2). Compared to the control, which had 70% egg hatching, both extracts, WE and EE, had a similar effect at 1 mg/mL and 10 mg/mL, ranging from 70% to 91% (Figure 2). Therefore, EE and WE did not have any toxic effect on egg hatching in the *C. elegans* model.

#### 3.3.3. *In Vivo* Antioxidant Activity Using the C*. elegans* Model

**Figure 3:**
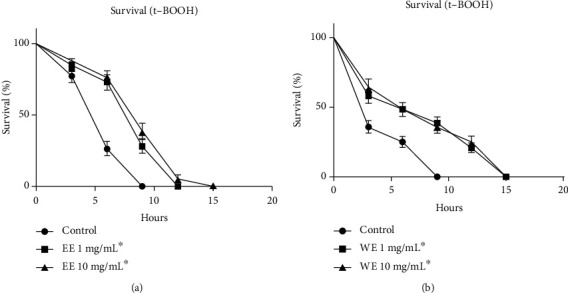
*In vivo* antioxidant activity of *C. alnifolia* extracts using the *C. elegans* model. (a) Survival assay of *C. elegans* worms treated with EE under oxidative stress treatment (t-BOOH). The EE treatment was with 0 mg/mL (control), 1 mg/mL, and 10 mg/mL (b) Survival assay of *C. elegans* worms treated with WE under oxidative stress treatment (t-BOOH). The WE treatment was with 0 mg/mL (control), 1 mg/mL, and 10 mg/mL. Survival for both treatments was observed every 3 h (3, 6, 9, 12, 15, 18, and 21 h). Worms were maintained at 20°C. The extract treatment was compared to the control by the Log-rank test (*p* < 0.0001). Data were collected in quintuplicate and were analysed by an ANOVA and the Tukey's test (*p* ≤ 0.05).

Because the EE and WE extracts did not have toxic effects on egg hatching and embryo development, these extracts were investigated for their *in vivo* antioxidant effects in *C. elegans*. For these assays, wild animals (N2) were used and were treated with EE and WE at 1 and 10 mg/mL. Subsequently, these worms were analysed for survival under oxidative stress conditions (treatment with *tert-butyl hydroperoxide* (t-BOOH)). An increased tolerance against oxidative stress conditions promoted by t-BOOH treatment was observed with EE and WE treatments (Figures 3(a) and 3(b)). For the control animals in the EE treatments, the average survival time was 7.5 h, compared with an average survival time of 10.5 h with EE (1 mg/mL and 10 mg/mL), indicating an increase of 28.6% (Table 3).

For the control animals in the WE treatments, the average survival time was 4.8 h compared with 7.9 h and 8.2 h for 1 mg/mL and 10 mg/mL WE, respectively (Figure 3(b)). This difference represented an increase of 39.2% (1 mg/mL) and 41.3% (10 mg/mL) under t-BOOH treatment (Table 4).

### 3.4. Phytochemical Compounds Identified by Thin-Layer Chromatography (TLC)

TLC analysis revealed that the presence of phenolic compounds such as flavonoids and their compounds may be associated with antioxidant activity observed in *in vitro* and *in vivo* assays. When the chromatoplates were revealed using sulphuric vanillin, colourful spots of saponins (CE, EE, ME, and WE), and terpenes (CE, EE, and ME) were observed. Furthermore, the chromatoplates in contact with ferric chloride solution developed spots that suggested the presence of phenolic compounds in the polar extracts (EE, ME, WEE, and EE), and with the Natural A Reagent, the presence of flavonoids in the polar extracts was observed (EE, ME, WEE, and WE). In order to identify some compounds present in polar active extracts, standards were used in co-TLC analysis, and vitexin (Rf = 0.51, green fluorescent) and isovitexin (Rf = 0.40, green fluorescent) were detected in the EE and ME. The presence of phenolic and flavonoids in all polar extracts, especially EE and ME, was observed.

### 3.5. WE Chemical Profile by HPLC-DAD

Based on the results observed with *in vitro* and *in vivo* assays for EE and WE and the traditional folk way of making extracts by maceration in water, we chose WE for further characterisation by high-performance liquid chromatography (HPLC-DAD), as TLC had shown the presence of vitexin and isovitexin.

**Figure 4:**
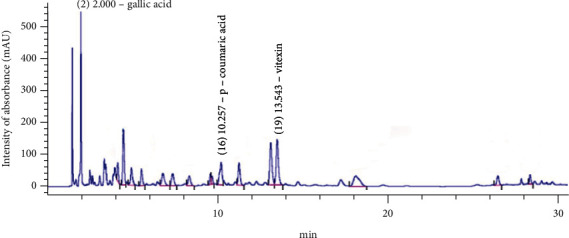
WE HPLC-DAD chromatogram. The WE analysis using HPLC-DAD. The 306 detection wavelength was set at 360 nm, the *x*-axis corresponds to the retention times (min) of each peak, and the *y*-axis corresponds to the intensity of the peaks in mAU. The peak absorption spectra #2 corresponds to gallic acid, #16 to *p*-coumarin, and #19 to vitexin.

The HPLC-DAD showed several peaks (Figure 4), which were evaluated at different UV spectra (228–352 nm). When compared to standards, peak #2 corresponded to gallic acid (tr = 2.000—280 nm), peak #16 corresponded to *p*-coumaric acid (tr = 10.257—280 nm), and peak #19 to vitexin (tr = 13.576—280-352 nm). The presence of vitexin was in agreement with the TLC data.

### 3.6. Bioinformatic Analysis

**Figure 5:**
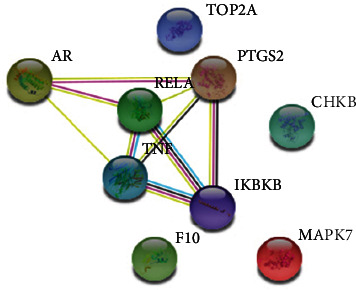
Schematic representation from an interactome using String 10 version 11. This interactome showed the possible gene targets from vitexin and isovitexin, phenolic compounds presented at *C. alnifolia* extracts. The data obtained from KEEG were used to build a network using String 10 version 11. Each circle corresponds to a gene; the lines indicate how these genes are connected. The blue lines indicate the curated database, pink lines indicate the determined experimentally, yellow lines indicate the data from text mining, black lines indicate the coexpression, and green line indicates a gene neighbourhood.

Bioinformatics is an excellent tool for data mining from potential targets considering the two bioactive molecules that were identified in the *C. alnifolia* extracts: vitexin and isovitexin. The Kyoto Encyclopedia of Genes and Genomes (KEGG) database was used to identify possible targets for these bioactive molecules. These molecules may be associated with pathways related to the immune system, cardiovascular disease, signalling pathways, cancer, nervous system, and others. More than 32 targets were observed (Table 5). When the overlap between targets was analysed, several gene targets such as IKBKB, PTGS2, RELA, TNF, MAPK7, AR, and NF-B were observed (Table 5 and Figure 5). Furthermore, the bioinformatics approach suggested that the bioactive molecules identified in *C. alnifolia* may have both antioxidant and anti-inflammatory activities.

## 4. Discussion

*Coccoloba alnifolia* has been used in folk medicine in northeastern Brazil, but there is no scientific information regarding its chemical composition or antioxidant activities. In this study, the chemical composition and biological activities of six leaf extracts were characterised using a series of solvents (apolar to polar serial approach) [32]. Chemical analyses showed the presence of sugars, proteins, and phenolic compounds. The proportion of these constituents varied among the extracts; EE, ME, WEE, and WE extracts contained high amounts of sugar and a moderate presence of phenolic compounds, whereas protein was very low in all the extracts.

Alam et al. [5] showed that polar solvents were good for extracting phenolic compounds, and the data presented here showed higher concentrations of phenolic compounds and flavonoids in these extracts (EE, WE, ME, WEE, and WE). TLC also showed the presence of terpenes (CE, EE, ME, and WE) and saponins (CE, EE, ME, and WEE). In co-TLC, the presence of vitexin and isovitexin was observed, while HPLC-DAD showed the presence of gallic acid, *p*-coumaric acid, and vitexin for WE. Although these compounds have been identified previously in other *Coccoloba* species [10, 33, 34], our findings are the first to report their presence in *C. alnifolia*. Based on this, it is important to evaluate the antioxidant activity and other biological activities of *C. alnifolia* extracts that contain a mixture of bioactive molecules besides vitexin and isovitexin, which were identified by co-TLC [35] and HPLC-DAD for WE.

The results obtained here showed excellent antioxidant potential for EE, ME, WEE, and WE, especially for reducing power and DPPH free radical scavenging. TAC showed higher antioxidant activity for EE and ME. DPPH evaluated the ability of a sample to scavenge DPHH free radicals, and both reducing power and TAC assays analysed the ability of the sample to donate electrons. Gusman et al. [34] observed antioxidant activity using the DPPH assay for *C. cereifera,* and antioxidant activity using DPPH has also been observed for other species from Polygonaceae such as *Rumex japonicus* [36] and *Polygonum maritimum* [37]. These species have been used in traditional medicine in Asia, Europe, and Africa, reinforcing the data obtained here for *C. alnifolia*. The antioxidant potential may be associated with phenolic compounds as well as sugars present in the extract, as shown by Pearson's correlations. The sugars identified may be associated with phenolic compounds that are linked to sugar molecules, such as flavonoid glycosides, condensed tannins, and glycoside triterpenes, which may explain the presence of sugars in *C. alnifolia* extracts [38, 39]. The antioxidant potential observed for these extracts may be associated with different bioactive molecules that might act to scavenge free radicals and reduce the ROS effects on cells, consequently maintaining the balance between production and degradation [35, 40, 41].

*In vitro* assays showed that *C. alnifolia* had interesting antioxidant potential, and *in vivo* assays using cell lines showed that, in general, the six extracts were neither cytotoxic to 3T3 nontumour cell lines nor to Hela, SiHa, PC-3, B16-F10, and PANC tumour cell lines. On the other hand, Tsuboy et al. [15] observed that root extracts from *C. mollis* were more cytotoxic than leaf extracts against HTC tumour cell lines. He et al. [42] showed that vitexin and isovitexin are excellent antioxidant molecules and have a wide spectrum of antioxidant, antiproliferative, anti-inflammatory, and other properties. Moreover, it has been verified that extracts from *C. uvifera* and *C. cereifera* have proinflammatory activity and may act on tumour necrosis factor-*α* (TNF-*α*) [33, 34]. The bioinformatics approach used here, considering the presence of vitexin and isovitexin, proposes that *C. alnifolia* extract may act on TNF-*α* and prostaglandin-endoperoxide synthase (PTGS or COX). Habtemariam [35] mentioned that extracts are a mixture of bioactive molecules that may act synergistically or alone. Furthermore, it has been observed that natural products may act on signalling pathways, for example, the NF-*κ*B signalling pathway [4, 35, 43–45]. Bioinformatics data using vitexin and isovitexin showed that some gene targets were anti-inflammatory genes such as NF-*κ*B and COX-2.

Moreover, *Caenorhabditis elegans* has been shown to be an excellent model for identifying toxicity in chemical compounds. There are some assays that may show toxicity, such as egg hatching and nematode development [46, 47]. The results obtained showed that EE and WE were not toxic because they did not affect egg hatching, and these extracts had a protective effect against oxidative stress when t-BOOH was used as a stressor agent. These extracts increased survival by 28% for 1 mg/mL EE, 31% for 1 mg/mL WE, and 42% for 10 mg/mL WE compared to that of the control animals. Yue et al. [48] observed that *p*-coumaric acid was able to reduce oxidative stress in *C. elegans* as well as increase its life span in oxidative stress conditions, similar to what was observed in EE and WE. Choubey et al. [49] showed different activities from gallic acid due to its antioxidant potential. Thus, *p*-coumaric acid, gallic acid, and vitexin may be some of the bioactive molecules present in our extract that have antioxidant potential and no toxicity, as observed in the data presented here for *Coccoloba alnifolia* extracts.

Other studies show phenolic compounds in the *Coccoloba* genus. Cota [10] working with *C. acrostichoides* identified betulin and *β*-sitosterol using TLC. Ashmawy et al. [12] working with *C. uvifera* leaves detected ellagic acid, benzoic acid, *o*-coumaric acid, rutin, myricetin, and quercetin in water, acetone, and ethanol extracts. Furthermore, for *C. mollis* triterpenes, diterpenes, anthraquinones, phytosteroids, and benzenes were identified in leaves and shoots [11].

For *C. alnifolia*, in the present study, phenolic compounds such as gallic acid, *p*-coumaric acid, vitexin, and isovitexin were observed, and HPLC-DAD showed that there are many other compounds to be identified. These extracts may have other bioactive molecules, which may act synergistically with phenolic compounds, resulting in oxidative protection, which reduces ROS effects and helps to maintain the redox balance (ROS production versus ROS degradation) in cells and tissues [35, 40]. The data presented here show that EE and WE have great potential as natural antioxidant products.

## 5. Conclusions

Five *Coccoloba alnifolia* leaf extracts were obtained by serial extraction (using apolar solvents to polar solvents), and the sixth extract was made with only water (based on traditional folk use). The data obtained here show that these extracts contain phenolic compounds, terpenes, saponins, and flavonoids (vitexin and isovitexin). In *in vitro* and *in vivo* assays, four polar extracts, EE, ME, WEE, and WE, were shown to be sources of antioxidant molecules. In general, these extracts did not affect the viability of six different mammalian cell lines. Moreover, EE and WE did not affect the fecundity of *Caenorhabditis elegans* nematodes used as a test model and protected them against the t-BOOH stressor, increasing their life span. Therefore, *Coccoloba alnifolia* leaves have excellent potential for the development of herbal medicines and as antioxidant products. Other biological activities may also be explored to determine how the potential antioxidants act in cells.

## Figures and Tables

**Table 1:** Total content of phenolics, proteins, and sugars in the extracts obtained from the leaves of *C. alnifolia.*

Extracts	Sugar (*μ*g/g)	Phenolic compounds (*μ*g/g)	Proteins (*μ*g/g)
Hexane (HE)	34.35	2.30	1.4
Chloroform (CE)	33.45	10.54	1.1
Ethanol (EE)	183.81	44.95	3.9
Methanol (ME)	225.00	61.26	4.5
Water end extract (WEE)	128.60	33.63	2.8
Water extract (WE)	198.56	17.67	2.0

**Table 2:** Effects of *C. alnifolia* extracts on cell viability using different concentrations (100, 250, and 500 *μ*g/mL) in nontumour and tumour cell lines after 24 h incubation.

Extracts	Conc.	Cell lines^#^					
		NIH/3T3	HeLa	SiHa	PC-3	B16-F-10	PANC-1
HE	100	120.68 ± 27.23	91.74 ± 19.92	75.59 ± 20.75	109.33 ± 23.12	139.64 ± 26.79	130.58 ± 40.12
	250	124.83 ± 44.05	112.44 ± 6.83	83.15 ± 12.66	113.08 ± 17.49	145.54 ± 23.01	162.30 ± 18.52
	500	188.97 ± 15.68	103.27 ± 1.77	95.68 ± 14.30	123.72 ± 27.13	104.56 ± 30.87	139.68 ± 28.42

CE	100	132.61 ± 3.41	74.26 ± 1.91	83.43 ± 7.66	117.07 ± 5.83	135.60 ± 83.95	113.97 ± 10.64
	250	131.22 ± 20.44	101.68 ± 12.80	98.37 ± 1.24	116.21 ± 11.43	206.72 ± 11.41	145.28 ± 10.74
	500	172.54 ± 15.33	99.22 ± 17.64	106.30 ± 3.93	132.68 ± 31.85	264.33 ± 63.63	158.97 ± 0.17

EE	100	130.19 ± 3.63	112.50 ± 37.64	105.33 ± 7.79	125.51 ± 1.86	84.69 ± 6.51	100.72 ± 6.31
	250	117.57 ± 28.21	119.26 ± 16.78	120.36 ± 20.10	125.12 ± 4.42	123.01 ± 18.46	118.93 ± 1.06
	500	100.45 ± 30.10	102.37 ± 30.54	122.24 ± 8.19	107.96 ± 10.68	120.18 ± 11.19	111.22 ± 14.92

ME	100	74.69 ± 22.23	88.57 ± 2.58	106.01 ± 1.04	122.33 ± 6.68	287.14 ± 91.82	138.11 ± 28.11
	250	67.25 ± 29.26	100.40 ± 3.89	118.88 ± 3.95	114.25 ± 7.24	270.75 ± 68.13	124.17 ± 7.75
	500	66.74 ± 6.32	74.26 ± 14.01	111.19 ± 10.36	99.12 ± 3.92	257.63 ± 60.13	137.70 ± 72.86

WEE	100	69.67 ± 76.06	95.81 ± 11.21	92.45 ± 31.21	125.24 ± 7.60	186.85 ± 76.03	126.62 ± 40.25
	250	67.08 ± 32.71	109.16 ± 30.89	106.87 ± 19.22	121.37 ± 2.20	214.59 ± 41.43	151.70 ± 55.60
	500	68.81 ± 28.83	83.49 ± 20.88	96.53 ± 22.22	100.87 ± 14.13	243.80 ± 139.33	145.63 ± 37.60

WE	100	109.96 ± 60.34	92.39 ± 30.05	103.31 ± 8.57	127.17 ± 32.17	133.90 ± 21.36	84.16 ± 4.91
	250	68.81 ± 34.60	91.78 ± 5.65	119.19 ± 12.50	109.21 ± 20.20	155.70 ± 50.79	89.09 ± 17.49
	500	70.37 ± 16.51	90.20 ± 0.13	99.35 ± 13.43	108.46 ± 7.45	170.46 ± 71.85	90.63 ± 17.25

HE: hexane extract; CE: chloroform extract; EE: ethanol extract; ME: methanol extract; WEE: water end extract; WE: water extract. ^#^NIH/3T3: ATCC CRL-1658—murine fibroblast; HeLa: ATCC CCL-2—adenocarcinoma cell of human uterus; SiHa: ATCC HTB-35—human squamous cellular carcinoma cell; PC-3: ATCC CRL-1435—human prostate adenocarcinoma cell; B16-F10: ATCC CRL-6475—mouse skin melanoma cell; PANC-1: ATCC CRL-1469—pancreatic adenocarcinoma cells.

**Table 3:** Survival assay using *C. elegans* worm to measure the effect of EE extract on the lifetime.

Survival condition	Mean of survival time (hours)	Variation of survival mean (%)	*p* value-Log-rank (Mantel-Cox) tests	Number of dead animals/censored
Control	7.5			80/0
EE 1 mg/mL	10.5	28.6	<0.0001	74/6
EE 10 mg/mL	10.5	28.6	<0.0001	80/0

**Table 4:** Survival assay using in vivo model—*C. elegans* model to evaluate the WE extract on lifetime.

Survival condition	Mean of survival time (hours)	Variation of survival mean (%)	*p* value-Log-rank (Mantel-Cox) tests	Number of dead animals/censored
Control	4.8			80/0
WE 1 mg/mL	7.9	39.2	<0.0001	80/0
WE 10 mg/mL	8.2	41.3	<0.0001	80/0

**Table 5:** KEGG pathways and gene targets after the overlap analysis for vitexin and isovitexin.

KEGG Brite (*Homo sapiens*)	Adjusted *p* value	Corrected *p* value	Related genes (targets)
Immune system	1.80*E*-12	2.36*E*-10	IKBKB;PTGS2;RELA;TNF;MAPK7
Signal transduction	1.37*E*-09	6.97*E*-08	IKBKB;PTGS2;RELA;TNF; MAPK7
Cardiovascular disease	4.96*E*-09	1.30*E*-07	IKBKB;RELA;TNF;MAPK7
Drug resistance: antineoplastic	1.68*E*-08	3.67*E*-07	IKBKB;RELA;TNF
Infectious disease viral	3.30*E*-08	6.17*E*-07	IKBKB;PTGS2;RELA;TNF
Infectious disease: bacterial	8.65*E*-07	5.67*E*-06	IKBKB;RELA;TNF
Infectious disease parasitic	2.05*E*-07	2.24*E*-06	PTGS2;RELA;TNF; IKBKB
Endocrine system	1.67*E*-07	2.08*E*-06	IKBKB;RELA;TNF
Signalling pathway	1.74*E*-07	2.08*E*-06	IKBKB;RELA;TNF
Development and Regeneration	1.02*E*-06	6.37*E*-06	IKBKB;RELA;TNF
Human disease: cancer	4.00*E*-07	4.03*E*-06	IKBKB;PTGS2;RELA; MAPK7
Endocrine and metabolic disease	6.20*E*-07	4.77*E*-06	IKBKB;RELA;TNF
Nervous system	8.24*E*-07	5.67*E*-06	IKBKB;RELA;MAPK7
Cell growth and death	1.22*E*-06	7.27*E*-06	IKBKB;RELA;TNF

## Data Availability

The data obtained in this study are available from the corresponding author upon request.

## Supplementary Materials

Supplementary Materials Supplementary Table 1 shows the Pearson coefficient analyses for sugar, protein, and phenolic compounds presented in each of the *Coccoloba alnifolia* extracts. The values obtained was shown in red, and it was considered a very strong correlation values higher than 0.9, strong correlation values from 0.7 to 0.9. The Pearson coefficient analyses showed a positive correlation between the phenolic compound content of the *Coccoloba alnifolia* extract and the antioxidant assay. There was a strong correlation between TAC and reducing power assay and a moderate correlation to superoxide radical scavenging. Furthermore, the sugar content presented a very strong correlation for reducing power assay and TAC and a moderate correlation with superoxide radical scavenging.
