# Supplementary material for: Coccoloba alnifolia Leaf Extract as a Potential Antioxidant Molecule Using In Vitro and In Vivo Assays
Source: Oxid Med Cell Longev. 2020 Oct 10;2020:3928706. doi: 10.1155/2020/3928706 (PMC7569468; doi:10.1155/2020/3928706)
Supplement: Supplementary Materials — Supplementary Table 1 shows the Pearson coefficient analyses for sugar, protein, and phenolic compounds presented in each of the Coccoloba alnifolia extracts. The values obtained was shown in red, and it was considered a very strong correlation values higher than 0.9, strong correlation values from 0.7 to 0.9. The Pearson coefficient analyses showed a positive correlation between the phenolic compound content of the Coccoloba alnifolia extract and the antioxidant assay. There was a strong correlation between TAC and reducing power assay and a moderate correlation to superoxide radical scavenging. Furthermore, the sugar content presented a very strong correlation for reducing power assay and TAC and a moderate correlation with superoxide radical scavenging. [file 3928706.f1.pdf]

Sup Table 1 - Pearson Correlation that was calculated considering the values obtained with antioxidant assays and sugar or to protein or to phenolic compounds

	**SUGAR**	TAC	DPPH	Superoxide	Reducing Power
CE	3.3	23.82	75.98	37.34	42.10
HE	3.4	11.03	78.43	77.48	16.18
WEE	12.9	55.38	84.64	75.06	99.56
EE	18.4	87.32	87.58	81.86	112.72
WE	19.9	40.67	68.14	75.06	108.33
ME	22.5	102.15	83.33	81.78	117.25
PEARSON		0.83	0.15	0.63	0.95

	**PROTEIN**	TAC	DPPH	Superoxide	Reducing Power
CE	0.1	23.82	75.98	37.34	42.10
HE	0.1	11.03	78.43	77.48	16.18
WEE	0.2	55.38	84.64	75.06	99.56
EE	0.3	87.32	87.58	81.86	112.72
WE	0.4	40.67	68.14	75.06	108.33
ME	0.5	102.15	83.33	81.78	117.25
PEARSON		0.78	0.01	0.55	0.84

	**PHENOLIC COMPOUNDS**	TAC	DPPH	Superoxide	Reducing Power
CE	0.23	23.82	75.98	37.34	42.10
HE	1.05	11.03	78.43	77.48	16.18
WEE	1.77	55.38	84.64	75.06	99.56
EE	3.36	87.32	87.58	81.86	112.72
WE	4.5	40.67	68.14	75.06	108.33
ME	6.13	102.15	83.33	81.78	117.25
PEARSON		0.77	0.05	0.63	0.79

Legend: very strong 0.9 or more; strong 0.7 to 0.9; moderate 0.5 to 0.7; weak 0.3 to 0.5; insignificant 0 to 0.3.

The supplementary Table 1 shown the Pearson coefficient analyzes for sugar, protein and phenolic compounds presented in each of the *Coccoloba alnifolia* extracts. It red it was shown the values obtained and it was considered a very strong correlation values higher than 0.9, strong correlation values from 0.7 to 0.9. The Pearson coefficient analyzes showed a positive correlation between the phenolic compounds content of the *Coccoloba*

alnifolia extract and the antioxidant assay. There was a strong correlation between TAC and Reducing Power Assay, and a moderate correlation to Superoxide Radical Scavening. Furthermore, the sugars content presented a very strong correlation for Reducing Power Assay and TAC and a moderate correlation with Superoxide Radical Scavening.
